# *Listeria monocytogenes* σ^A^ Is Sufficient to Survive Gallbladder Bile Exposure

**DOI:** 10.3389/fmicb.2019.02070

**Published:** 2019-09-04

**Authors:** Atsadang Boonmee, Haley F. Oliver, Soraya Chaturongakul

**Affiliations:** ^1^Department of Microbiology, Faculty of Science, Mahidol University, Bangkok, Thailand; ^2^Department of Food Science, College of Agriculture, Purdue University, West Lafayette, IN, United States

**Keywords:** RNA-seq, *Listeria monocytogenes*, housekeeping sigma σ^A^, sigma factor, bile, stress

## Abstract

*Listeria monocytogenes* is a foodborne Gram-positive bacterium causing listeriosis in both animals and humans. It can persist and grow in various environments including conditions countered during saprophytic or intra-host lifestyles. Sigma (σ) subunit of RNA polymerase is a transcriptional factor responsible for guiding the core RNA polymerase and initiating gene expression under normal growth or physiological changes. In *L. monocytogenes*, there is one housekeeping sigma factor, σ^A^, and four alternative sigma factors σ^B^, σ^C^, σ^H^, and σ^L^. Generally, σ^A^ directs expression of genes required for normal growth while alternative σ factors alter gene expression in response to specific conditions (e.g., stress). In this study, we aimed to determine the exclusive role of σ^A^ in *L. monocytogenes* by comparing a wild type strain with its isogenic mutant lacking genes encoding all alternative sigma factors (i.e., *sigB*, *sigC*, *sigH*, and *sigL*). We further investigated their survival abilities in 6% porcine bile (pH 8.2) mimicking gallbladder bile and their transcriptomics profiles in rich medium (i.e., BHI) and 1% porcine bile. Surprisingly, the results showed that survival abilities of wild type and Δ*sigB*Δ*sigC*Δ*sigH*Δ*sigL* (or Δ*sigBCHL*) quadruple mutant strains in 6% bile were similar suggesting a compensatory role for σ^A^. RNA-seq results revealed that bile stimulon of *L. monocytogenes* wild type contained 66 genes (43 and 23 genes were up- and down-regulated, respectively); however, only 29 genes (five up- and 24 down-regulated by bile) were differentially expressed in Δ*sigBCHL*. We have shown that bile exposure mediates increased transcription levels of *dlt* and *ilv* operons and decreased transcription levels of *prfA* and heat shock genes in wild type. Furthermore, we identified σ^A^-dependent bile inducible genes that are involved in phosphotransferase systems, chaperones, and transporter systems; these genes appear to contribute to *L. monocytogenes* cellular homeostasis. As a result, σ^A^ seemingly plays a compensatory role in the absence of alternative sigma factors under bile exposure. Our data support that the bile stimulon is prone to facilitate resistance to bile prior to initiated infection.

## Introduction

The foodborne pathogen *Listeria monocytogenes* is a facultative Gram-positive intracellular bacterium that is able to adapt to a broad range of environments such as soil and waste water. It can survive and grow in a variety of temperature ranging from −1.5°C to up to 45°C, a wide range of pH and hypertonic conditions (Hudson et al., 1994; Membre et al., 2005; Goulet et al., 2013). This pathogen is the causative agent of listeriosis in human and animals. Immunocompromised individuals, including the elderly and pregnant women, are considered to be high-risk populations (Southwick and Purich, 1996). While *L. monocytogenes* infections are rare, the mortality rate is 15.6% (EFSA, 2015). It ranks third among foodborne pathogens causing death in the United States (Scallan et al., 2011) and results in approximately 23,000 cases of listeriosis each year worldwide (de Noordhout et al., 2014).

Sigma factors (σ) are the dissociable subunit of bacterial RNA polymerase (RNAP) enzyme; they recognize specific promoter sequences and initiate gene/operon transcription. Unbound sigma factors can bind to core RNAP to form the holoenzyme and enhance interaction between RNAP and promoter consensus sequences (Burgess, 2001). This results in conformational changes in the recognized promoter regions upstream of the transcription starting site. Most gene expression in the bacterial cells is dependent on the housekeeping sigma factor σ^70^ in *Bacillus subtilis* (Gruber et al., 2001) or σ^A^ in *L. monocytogenes*. σ^70^ is encoded by *rpoD* in *E. coli* and it is evident that the intracellular concentrations of RNAP and RpoD remain constant under several growth conditions (Jishage et al., 1996; Piper et al., 2009). σ^70^ is required for cell growth related transcription, while alternative sigma factors regulate gene expression in response to stress (Mauri and Klumpp, 2014). For example, the alternative sigma factor σ^B^ is known to respond to heat stress in *L. monocytogenes* and *B. subtilis* (Schumann, 2003) as well as σ^*F*^, σ^*E*^, σ^*G*^, and σ^*K*^ are known for developmental programing such as sporulation in *B. subtilis* (Losick and Stragier, 1992). The concentration of alternative sigma factors [e.g., σ^*H*^ (σ^32^), σ^*S*^ (σ^38^) in *E. coli*] vary considerably within altered physiological states (Jishage et al., 1996; Grigorova et al., 2006).

Under different environmental conditions, *L. monocytogenes* regulates gene expression through the use of four alternative sigma factors: σ^B^, σ^C^, σ^*H*^, and σ^*L*^ (Chaturongakul et al., 2011). Co-regulation between σ^A^ and each alternative sigma factor have been evaluated. A number of genes have σ^A^- and σ^B^-dependent promoters such as *prfA* (encoding a master regulator of virulence genes), *qoxABCD* (encoding quinol oxidase important for oxidative stress response), and *cggR* (encoding central glycolytic gene regulator) (Liu et al., 2017). Promoter consensus sequences of σ^A^ and σ^*H*^ have been found in competence genes such as *comG* (Medrano Romero and Morikawa, 2016). Genes co-regulated by housekeeping σ^A^ and other alternative sigma factors have also been identified in other bacteria including *B. subtilis* and *Escherichia coli* (Wade et al., 2006). The extracytoplasmic function (ECF) sigma factor is also shown to co-regulate with σ^A^ in *B. subtilis* (Kingston et al., 2011). In addition to the concentration of sigma factors inside the cell, the affinity between each sigma factor and RNAP determines the probability of associating with the core RNAP. In *E. coli*, σ^70^ has been shown to have the highest affinity with core RNAP (Sharma and Chatterji, 2010). Previous studies have demonstrated that sigma factors can compensate for each other as explored in *in vitro* transcriptional assays (Maeda et al., 2000; Jishage et al., 2002) and competition experiments (Mauri and Klumpp, 2014).

A *L. monocytogenes* quadruple deletion mutant (Δ*sigB*Δ*sigC*Δ*sigH*Δ*sigL* or Δ*sigBCHL*) with only σ^A^ remaining grew as well as wild type in phosphotransferase system (PTS)-dependent carbon sources (e.g., mannose, cellobiose, and glucose) (Wang et al., 2014) suggesting that having the housekeeping σ^A^ alone is sufficient to maintain transcription. This raised our interest in the potential of up-regulation of genes by σ^A^ in stress conditions in *L. monocytogenes*. For instance, following consumption of contaminated food, *L. monocytogenes* encounters the low pH of the stomach and the bile salt and high osmolality in intestinal fluid during gastric passage. In order to colonize the intestine and successfully establish infection, *L. monocytogenes* needs to survive bile exposure, an important antimicrobial component in gastrointestinal fluid (Olier et al., 2004). It has also been reported that *L. monocytogenes* may utilize unique bile resistance mechanisms to survive in the gallbladder (Hardy et al., 2004). A number of studies have evaluated *L. monocytogenes* proteins that affect bile resistance including bile salt hydrolase (Bsh) (Dussurget et al., 2002; Begley et al., 2005b), the bile exclusion system (BilE) (Sleator et al., 2005), and multidrug resistance (MDR) efflux pump MdrT (Quillin et al., 2011). Previously, it was determined that bile salt hydrolase as well as bile exclusion system are regulated by σ^B^ (Dussurget et al., 2002; Begley et al., 2005b). However, the bile stimulon defined recently by RNA-seq analyses was not identified as σ^B^-dependent (Guariglia-Oropeza et al., 2018). This is surprising, however, they suggested that it is possibly due to comparing transcriptional profiles of *L. monocytogenes* strains exposed to pH 5.5 with and without bile. The σ^B^ regulon is induced under acidic condition (Sue et al., 2004); no induction has been observed in the presence of bile. It is more likely that σ^B^-dependent gene expression plays a more crucial role in earlier stage of gastrointestinal infection (e.g., stomach) and prime the bacteria for the establishment in the small intestine. We hypothesized that under bile stress exposure, Δ*sigBCHL* will have similar survival ability as wild type due to the compensatory role of housekeeping σ^A^ in Δ*sigBCHL*. To test the hypothesis, we phenotypically and transcriptomically compared Δ*sigBCHL* with solely functional σ^A^ with its isogenic parental wild type under bile exposure. Genes under σ^A^ regulation in *L. monocytogenes* during gallbladder bile exposure were identified.

## Materials and Methods

### Bacterial Strains and Growth Conditions

*Listeria monocytogenes* 10403S and its isogenic quadruple alternative sigma factor mutant strain, Δ*sigBCHL* [FSL C3-135 (Mujahid et al., 2013)] were used in this study. Both strains were maintained in Brain Heart Infusion broth (BHI; Difco^TM^, BD, United States) and 50% glycerol stocks at −80°C. They were streaked onto BHI agar plates prior to each experiment, and plates were incubated at 37°C overnight. A single colony inoculated into five ml BHI broth for 37°C overnight (16–18 h) incubation with shaking (200 rpm). Overnight culture was diluted 1:100 into a fresh five ml BHI broth and incubated at 37°C with shaking to an OD_600_ of approximately 0.4, representing mid-log phase. An aliquot of 500 μl of the culture was subsequently passaged in 50 ml of BHI broth to generate synchronized cells at mid-log phase before exposure to the simulated bile stress.

### Bile Stress Survival Assay

Once mid-log phase was reached, cells were immediately challenged with simulated gallbladder bile. The *in vitro* bile fluid was prepared as previously described (Versantvoort et al., 2005). Briefly, 0.5 ml of 2 × 6% porcine bile (Sigma, United States), pH 8.2 ± 0.2 (30 ml of 175.3 g/l NaCl, 68.3 ml of 84.7 g/l NaHCO_3_, 4.2 ml of 89.6 g/l KCl, 150 μl of 37% g/g HCl, 10 ml of 25 g/l urea, 10 ml of 22.2 g/l CaCl_2_.2H2O, and 1.8 g BSA to 500 ml distilled water) was added to 0.5 ml of mid-log phase culture. The challenged cultures were incubated at 37°C with shaking for 10 and 20 min, respectively. Survival ability was determined at 10 and 20 min after stress (*t* = 10 and *t* = 20). A 100 μl aliquot of the pre-treated control, untreated control (culture with additional 20 min incubation) along with *t* = 10 and *t* = 20 cultures were ten-fold serially diluted in PBS (pH 7.4); 10 μl of each dilution was plated onto BHI agar plates for subsequent enumeration. Experiments were conducted in triplicate on separate days.

### Statistical Analysis for Survival Assay

The significant differences in survival between *L. monocytogenes* wild type and quadruple mutant were determined by *t*-test (SPSS v. 23, IBM, United States). *p* < 0.05 was considered statistically significantly different.

### RNA Isolation and DNase Treatment

RNA was extracted from mid-log phase cultures after 10 min exposure to 1% porcine bile or BHI (as a control) as previously described with minor modifications (Pleitner et al., 2014). Briefly, all experiments were conducted in three biological replicates on different days. For each strain and condition, one ml of BHI or bile-exposed culture was collected and immediately added to ice-cold stop solution of 10% acid-phenol chloroform pH 4.5 (Invitrogen^TM^, United States) in ethanol, and followed as outlined previously (Pleitner et al., 2014). DNase treatment was performed using TURBO DNA-*free*^TM^ DNase treatment kit (Invitrogen, United States) according to the manufacturer’s protocol. Total RNA concentration was quantified using Nanodrop (DeNovix, United States).

### rRNA Depletion, Library Preparation, and RNA Sequencing

Ribosomal RNA (rRNA) was depleted by using Ribo-Zero rRNA removal kit (Epicentre, Madison, WI, United States) following manufacturer’s instruction. Quality of RNA was assessed using the 2100 Bioanalyzer (Agilent Technology, Santa Clara, CA, United States). Sample with RNA Integrity Number (RIN) score greater than 8.0 was considered acceptable for further library preparation and RNA-sequencing.

cDNA library preparation was constructed using ScriptSeq v2 RNA-seq Library Preparation kit for Bacteria (Epicentre, Madison, WI, United States). Purification of cDNA and indexed RNA-seq libraries were performed using Agencourt^®^ AMPure^®^ XP kit (Beckman Coulter Inc., Brea, CA, United States). Quantity and quality of the libraries were determined using the 2100 Bioanalyzer. All experiments were performed in three biological replicates. Sequencing was carried out on a HiSeq 2 × 100 High Output paired-end, 100 bp read at the Purdue University genomics core facility.

### RNA-Seq Analysis

Sequencing reads were mapped against *L. monocytogenes* 10403S (NCBI accession number: NC_017544.1). The average coverage per base on both sense and anti-sense strands of *L. monocytogenes* 10403S wild type and quadruple mutant (Δ*sigBCHL*) as well as rRNA match rates in BHI and bile are shown in Supplementary Table S1. The paired-end RNA-seq data were submitted to the SRA database (SRA accession number: PRJNA544468). Reads were aligned and mapped with Bowtie2 and TopHat2 (Langmead and Salzberg, 2012; Trapnell et al., 2012). Reads were counted by HTSeq-count (Anders et al., 2015). Differential expression (DE) was compared and analyzed in R version 3.3.3 using the package DESeq2 (Love et al., 2014). The analyses were conducted from three replicates of each sample, except wild type sample in BHI (two replicates were used in analysis). Genes were considered differentially expressed when log_2_ fold-change <−1 or >1 (representing down- or up-regulation) and adjusted *p*-values < 0.05.

### Gene Set Enrichment Analysis

GOseq package 1.34.1 package for R (Young et al., 2010) available from Bioconductor was used to evaluate whether differential expressed genes identified by DESeq2 were enriched for Gene Ontology (GO) terms. This tool allowed us to statistically confirm the specific metabolic pathways that *L. monocytogenes* utilized under BHI and/or bile conditions.

### Quantitative PCR (qPCR) Validation of RNA-Seq Data

Selected differentially expressed genes from RNA-seq results were validated using qPCR as previously described with minor modifications (Sue et al., 2004; Pleitner et al., 2014). Target genes used for RNA-seq data validation were (i) *rpoB* and *bglA* as housekeeping genes and (ii) *gadT2*, *plcA*, *LMRG_00091*, *LMRG_01149*, *LMRG_01669*, and *LMRG_02283* for sigma A function. A list of TaqMan primers and probes designed by PrimerQuest (IDT DNA, Coralville, IA, United States) are shown in Table 1. TaqMan probes were synthesized with a 5′ 6-carboxyfluorescein (6-FAM) reporter dye and a 3′ ZEN^TM^ dark quencher dye. All reactions were run via Rotor-Gene Q (Qiagen, Germany). cDNA synthesis was performed at 48°C for 30 min followed by PCR setting of 1 cycle at 95°C for 10 min, 40 cycles at 95°C for 15s, and 55°C for 1 min. Transcripts were normalized by a geometric mean of housekeeping genes, *rpoB* and *bglA*. The transcript levels of each strain in BHI and bile were compared statistically using unpaired *t*-test, SPSS version 23 (IBM, United States). Statistical comparison of gene expression levels among *L. monocytogenes* strains in each condition were analyzed by unpaired *t*-test.

**TABLE 1:** TaqMan primers and probes used for RNA-seq data validation.

**Target gene**	**Forward primer^∗^**	**Probe^∗^**	**Reverse primer^∗^**
*bglA*	TTCATGAGCGGCGGTATTT	TACCAAGCTGTCCACCACGAACTT	TGTGCTTCGGGCATGATT
*gadT2*	TGGCAAGAAGGCGGTATTT	TTCTTGGGTGGGAAATACACTCGGG	TCACGAATCCGACCGTTATTT
*malG*	GCAGCCCTAACAGCTTTCT	AGCAATGCTACTAGGTGCGCTTGA	GAATCCCACCACCGATGTAAA
*plcA* Kazmierczak et al., 2006	GATTTATTTACGACGCACATTAGTTT	CCCATTAGGCGGAAAAGCATATTCGC	GAGTTCTTTATTGGCTTATTCCAGTTATT
*rpoB* Pleitner et al., 2014	CGATCTTGGAGAGCCGAAATA	CGGTAGAAGAATCTAAGAAC	GAGCCGCATAGTTTGCATCAC
*LMRG_01149*	GAGCATCTATCCCTCCAGAAATTA	TAGGAACTGCCTTCGCGATTTCGA	AATCCCAAGAGCTAACGCTAC
*LMRG_01669*	GATTTCGCAAAGACTCGGATTAC	TTGCACCAAGTTTGCGAGAATGGC	ACGACGACGAATCGCTTT
*LMRG_02283*	GTCATTTACCAGCCCGTATCTC	AGTACCGCTTGTTTGGTCAATTTGGT	CGGACTCATCATGTTCCAATCA

*^∗^ All sequences are written 5′–3′.*

## Results

### *L. monocytogenes* Lacking All Alternative Sigma Factors Has Similar Phenotype as Its Parental Strain

**FIGURE 1:**
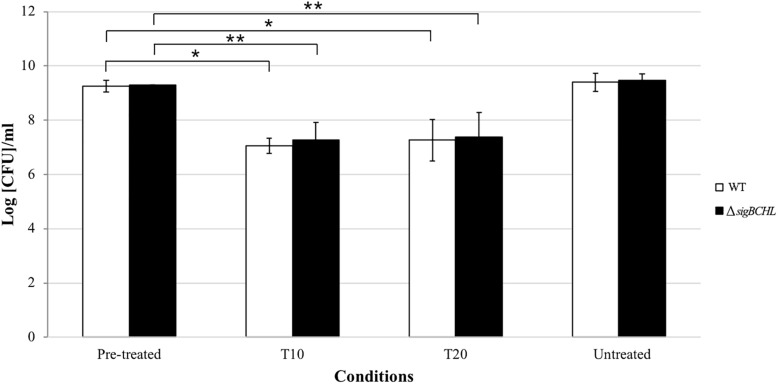
Survival of *Listeria monocytogenes* 10403S wild type and its quadruple mutant in simulated gallbladder bile at various time points: pre-treated control, T10, T20, untreated control; WT (white), Δ*sigBCHL* (black). Asterisks indicate significant difference when compared to pre-treated controls using *t*-test (*p* < 0.05).

To examine the survival ability under bile exposure, *L. monocytogenes* wild type and the quadruple mutant (Δ*sigBCHL*) were exposed to simulated gallbladder bile for 20 min. Viability of each culture was determined at 10 and 20 min after bile treatment; two log CFU/ml reductions were observed in both wild type and Δ*sigBCHL* mutant when compared to untreated controls (*p* < 0.05) (Figure 1). No significant difference was observed between wild type and Δ*sigBCHL* mutant suggesting housekeeping sigma factor σ^A^ alone is sufficient to coordinate expression of genes responsible for survival under bile stress.

### Sixty-Six Significantly Differentially Expressed Genes Were Identified in Wild Type *L. monocytogenes* Exposed to Bile

**FIGURE 2:**
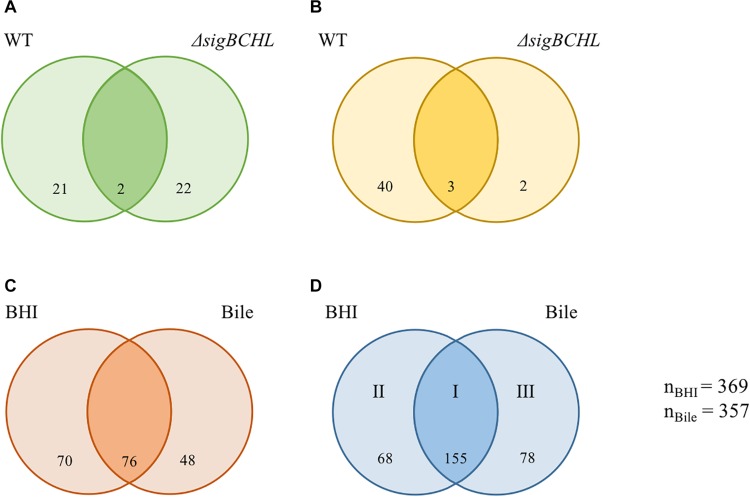
Venn diagrams showing the overlaps of differentially expressed genes (DEGs) of *L. monocytogenes* wild type and Δ*sigBCHL* under bile exposure compared to BHI. **(A,B)** Numbers of genes with significantly decreased and increased transcripts after exposure to bile, respectively. **(C,D)** Numbers of genes under σ^A^ regulation with significantly lower and higher levels in Δ*sigBCHL* in comparison to wild type, respectively. Differentially expressed genes in BHI and bile conditions are shown in left and right circles, respectively. The number indicated in the circle represents the number of differentially expressed genes in each strain and condition. I, II, and III represent groups of genes positively regulated by σ^A^. The total numbers of differentially expressed genes under BHI and bile calculated from either lower or higher level are 369 and 357, respectively.

We evaluated wild type and Δ*sigBCHL* mutant transcriptomic profiles in both BHI and bile conditions to characterize the bile stimulon and σ^A^ regulon. To define the bile response genes of *L. monocytogenes* 10403S, we compared RNA-seq data from wild type grown in BHI (control) to 1% bile for 10 min. Genes with FC either >2 or <−2 and adjusted *p*-value < 0.05 were considered significant difference. We found 66 genes significantly differentially expressed upon bile exposure of which 23 and 43 genes were down- and up-regulated, respectively (Figures 2A,B and Table 2). Twenty-three genes with statistically significant reduced transcript levels were decreased by two–three folds. Among these genes, *purE*, *LMRG_02276*, and *groL* were the most down-regulated with FC of 3.3, 3.2, and 3.2, respectively. *purE* encodes a phosphoribosylaminoimidazole carboxylase catalytic subunit involved in inosine-5′-phosphate biosynthesis II. *LMRG_02276* and *groL* encode QacE family quaternary ammonium compound efflux SMR transporter and heat shock protein 60 family chaperone (GroL), respectively. Surprisingly, the master positive virulence regulator, PrfA, was down-regulated (FC = 2.14) in bile exposure conditions. The chaperone encoding gene *dnaK* was also shown to have lower transcript levels after bile exposure. *opuCA* and *opuCD* involved in L-carnitine/choline ABC transporter were also down-regulated. On the other hand, among the up-regulated genes, *LMRG_01622* had the highest fold-change at 18.59; its function is not yet characterized. We also observed that *inlC2* (encoding internalin C2), *dltC* (encoding D-alanyl carrier protein), *ilvBCD* (encoding valine biosynthesis associated proteins), and *uspA2* (encoding universal stress protein) were up-regulated under bile exposure. GO terms associated with biological process involved in defense response, antigenic variation, and interspecies interaction between organisms were enriched in bile exposure condition (Supplementary Table S2).

**TABLE 2:** Differentially expressed genes under bile exposure in *L. monocytogenes* 10403S wild type.

***LMRG* Locus tag**	***lmo* Locus tag**	**Gene name**	**Operon**	**Annotation**	**FC**	**Adjusted *p*-value**
*LMRG_00151*			*LMRG_02887*, *LMRG_00151- LMRG_00152*	Hypothetical protein	3.08	0.00
*LMRG_00152*				Hypothetical protein	2.39	0.01
*LMRG_00222*	*lmo0540*			Penicillin-binding protein	2.04	0.00
*LMRG_00293*	*lmo0610*			Internalin-like protein	2.4	0.00
*LMRG_00311*	*lmo0628*		*LMRG_00311-LMRG_00312*	Hypothetical protein	4.56	0.00
*LMRG_00334*	*lmo0647*			Hypothetical protein	2.83	0.00
*LMRG_00341*	*lmo0654*		*LMRG_00341-LMRG_00342*	Hypothetical protein	3.11	0.00
*LMRG_00404*	*lmo0715*		*LMRG_00402-LMRG_00407*	Hypothetical protein	2.02	0.00
*LMRG_00482*	*lmo0794*			Putative Rrf2-linked NADH-flavin reductase	2.41	0.00
*LMRG_00529*	*lmo1067*			GTP-binding protein TypA/BipA	2.23	0.00
*LMRG_00530*	*lmo1068*			Fomain-containing protein	2.05	0.01
*LMRG_00706*	*lmo1257*			Hypothetical protein	2.53	0.00
*LMRG_00826*	*lmo1375*			Peptidase M20	2.07	0.00
*LMRG_00942*	*lmo1489*		*LMRG_00945-LMRG_00939*	RNA binding protein	2.22	0.00
*LMRG_01131*	*lmo1983*	*ilvD*	*ilv-leu*	Dihydroxy-acid dehydratase	2.62	0.01
*LMRG_01132*	*lmo1984*	*ilvB*		Acetolactate synthase large subunit	3.12	0.00
*LMRG_01134*	*lmo1986*	*ilvC*		Ketol-acid reductoisomerase	2.91	0.00
*LMRG_01453*	*lmo1517*		*LMRG_01454-LMRG_01453*	Nitrogen regulatory protein P-II	2.07	0.02
*LMRG_01561*	*lmo2269*			Hypothetical protein;	6.18	0.00
*LMRG_01602*	*lmo2230*	*arsC*	*Ars*	Arsenate reductase	2.56	0.01
*LMRG_01609*	*lmo2223*			Putative transcriptional regulator	2.09	0.04
*LMRG_01622*	*lmo2210*			Hypothetical protein	18.59	0.00
*LMRG_01630*	*lmo2202*	*fabH*	*LMRG_01630-LMRG_01631*	3-oxoacyl-[acyl-carrier-protein] synthase, KASIII	2.05	0.00
*LMRG_01761*	*lmo2487*			Hypothetical protein	2.14	0.00
*LMRG_01976*	*lmo2720*			Acyl-coenzyme A synthetases/AMP-(fatty) acid ligases, YtcI homolog	4.32	0.00
*LMRG_02011*	*lmo0911*			Hypothetical protein	2.43	0.00
*LMRG_02071*	*lmo0972*	*dltC*	*dltABCD*, *LMRG_02074*	D-alanyl carrier protein	2.04	0.00
*LMRG_02074*				Teichoic acid D-Ala incorporation-associated protein DltX	2.28	0.01
*LMRG_02191*	*lmo2646*		*LMRG_02193-LMRG_02190*	Hypothetical protein	2.51	0.03
*LMRG_02218*	*lmo2673*	*uspA2*	*uspA2-rpiB*	Universal stress protein UspA	2.37	0.02
*LMRG_02232*	*lmo2686*			Hypothetical protein	2.23	0.00
*LMRG_02304*	*lmo0880*			Putative peptidoglycan bound protein (LPXTG motif)	4.47	0.00
*LMRG_02364*	*lmo0115*	*lmaD*	*lmaDCBA*	*Listeria* protein LmaD, associated with virulence	3.79	0.00
*LMRG_02365*	*lmo0116*	*lmaC*		LmaC, associated with virulence in *Listeria*	3.34	0.00
*LMRG_02366*	*lmo0117*	*lmaB*		*Listeria* protein LmaB, associated with virulence	2.04	0.02
*LMRG_02372*	*lmo0123*		*LMRG_02369-LMRG_02378*	Putative tail or base plate protein gp18 [Bacteriophage A118]	2.1	0.04
*LMRG_02423*	*lmo2852*			ASCH domain-containing protein	2.02	0.02
*LMRG_02427*	*lmo2856*	*rpmH*		50S ribosomal protein L34	2.06	0.00
*LMRG_02611*	*lmo0265*	*dapE*		Succinyl-diaminopimelate desuccinylase	2.87	0.00
*LMRG_02646*	*lmo0263*	*inlC2*, *inlH*		Internalin C2	2.22	0.04
*LMRG_02700*	*lmo2568*		*LMRG_02700-LMRG_02701*	Hypothetical protein	9.41	0.00
*LMRG_02768*	*lmo1694*			CDP-abequose synthase, Putative sugar nucleotide epimerase	3.68	0.00
*LMRG_02808*	*lmo2132*			Cyclic nucleotide-binding protein	2.52	0.00
*LMRG_00273*	*lmo0591*		*LMRG_00271-LMRG_00273*	Hypothetical protein	−2.12	0.01
*LMRG_00501*	*lmo1040*		*LMRG_00501-LMRG_00499*	molybdenum ABC transporter permease	−2.57	0.04
*LMRG_00852*	*lmo1400*		*LMRG_00852-LMRG_00854*	Phosphinothricin N-acetyltransferase	−2.20	0.00
*LMRG_00877*	*lmo1425*	*opuCD*	*opuC*	Osmotically activated L-carnitine/choline ABC transporter, permease protein OpuCD, subunit of predicted ATP-driven transporter complex of CARNITINE/choline	−2.29	0.00
*LMRG_00880*	*lmo1428*	*opuCA*		Osmotically activated L-carnitine/choline ABC transporter, ATP-binding protein OpuCA, subunit of predicted ATP-driven transporter complex of CARNITINE/choline	−2.05	0.00
*LMRG_00926*	*lmo1473*	*dnaK*	*hrcA-grpE-dnaK-dnaJ*	Chaperone protein, DnaK	−2.39	0.00
*LMRG_01023*	*lmo1877*	*fhs*		Formate–tetrahydrofolate ligase	−2.55	0.00
*LMRG_01218*	*lmo2068*	*groL*	*groSL*	Heat shock protein 60 family chaperone GroEL	−3.20	0.00
*LMRG_01219*	*lmo2069*	*groS*		Heat shock protein 60 family co-chaperone GroES	−2.58	0.00
*LMRG_01286*	*lmo1681*		*LMRG_01286-LMRG_01288*	5-methyltetrahydropteroyltriglutamate–homocysteine methyltransferase	−2.03	0.03
*LMRG_01399*	*lmo1568*		*LMRG_01399-LMRG_01401*	Membrane protein	−2.46	0.00
*LMRG_01585*	*lmo2247*		*LMRG_01585-LMRG_01587*	Xidoreductase of aldo/keto reductase family, subgroup 2, glyoxal reductase	−2.09	0.01
*LMRG_01842*	*lmo2406*			Hypothetical protein	−2.38	0.00
*LMRG_02144*	*lmo2600*		*LMRG_02142-LMRG_02144*	ATPase component of general energizing module of ECF transporters	−2.40	0.00
*LMRG_02241*	*lmo2694*			Arginine decarboxylase/Lysine decarboxylase	−2.14	0.00
*LMRG_02276*	*lmo0853*		*LMRG_02275-LMRG_02277*	QacE family quaternary ammonium compound efflux SMR transporter	−3.22	0.02
*LMRG_02326*	*lmo0075*		*LMRG_02326-LMRG_02327*	Phosphonomutase, probable carboxyvinyl-carboxyphosphonate phosphorylmutase	−2.03	0.04
*LMRG_02358*	*lmo0109*		*LMRG_02358-LMRG_02359*	AraC family transcriptional regulator	−2.18	0.00
*LMRG_02386*	*lmo0137*		*LMRG_02385-LMRG_02386*	Oligopeptide ABC transporter, permease protein	−2.29	0.03
*LMRG_02496*	*lmo1775*	*purE*	*LMRG_02496-LMRG_02507*	Phosphoribosylaminoimidazole carboxylase catalytic subunit, 5-(carboxyamino)imidazole ribonucleotide mutase	−3.30	0.00
*LMRG_02510*	*lmo1761*			Sodium-dependent transporter	−2.78	0.00
*LMRG_02622*	*lmo0200*	*prfA*	*prfA-plcA*	Listeriolysin regulatory protein, PrfA	−2.14	0.00
*LMRG_02716*	*lmo2371*		*LMRG_02716-LMRG_02717*	ABC transporter, permease protein	−2.59	0.00

### Twenty-Nine Significantly Differentially Expressed Genes Were Identified in *L. monocytogenes* Quadruple Mutant Exposed to Bile

In the Δ*sigBCHL* strain, we were able to assess the role σ^A^ alone plays in response to bile. To assess this assumption, Δ*sigBCHL* RNA-seq data from exposed and non-exposed to bile were compared for differentially expressed genes. Remarkably, we found a small number of differentially expressed genes in this comparison; in total, 29 genes were identified. Of these, 24 and five genes were down- and up-regulated under bile treatment, respectively. Overall, 2–3 FC was observed (Table 3). No specific GO terms were enriched among these genes. Among the five bile up-regulated genes, four encode hypothetical proteins and only *LMRG_02808* is annotated as a cyclic nucleotide-binding protein.

**TABLE 3:** Differentially expressed genes under bile exposure in *L. monocytogenes*Δ*sigBCHL* quadruple mutant.

**LMRG Locus tag**	**lmo Locus tag**	**Gene name**	**Operon**	**Annotation**	**FC**	**Adjusted *p*-value**
*LMRG_00151*			*LMRG_02887*, *LMRG_00151-LMRG_00152*	Hypothetical protein	2.23	0.00
*LMRG_01622*	*lmo2210*			Hypothetical protein	4.25	0.00
*LMRG_01645*	*lmo2187*			Hypothetical protein	2.22	0.02
*LMRG_01919*	*lmo2778*			Hypothetical protein	2.05	0.03
*LMRG_02808*	*lmo2132*			Cyclic nucleotide-binding protein	2.08	0.00
*LMRG_00091*	*lmo0398*		*LMRG_00091-LMRG_00095*	PTS system, IIA component	−2.51	0.01
*LMRG_00092*	*lmo0399*			PTS system, IIB component	−3.52	0.00
*LMRG_00352*	*lmo0665*		*LMRG_00351-LMRG_00353*	Hypothetical protein	−2.32	0.01
*LMRG_00353*	*lmo0666*			Domain-containing protein	−2.12	0.01
*LMRG_00369*	*lmo0681*			Flagellar biosynthesis regulator FlhF	−2.01	0.00
*LMRG_00373*	*lmo0685*	*motA*	*motAB*	Flagellar motor rotation protein MotA	−2.43	0.00
*LMRG_00852*	*lmo1400*		*LMRG_00852-LMRG_00854*	phosphinothricin N-acetyltransferase	−2.06	0.00
*LMRG_01073*	*lmo1926*		*LMRG_01075-LMRG_01070*	Chorismate mutase II	−2.2	0.01
*LMRG_01156*	*lmo2008*		*LMRG_01157-LMRG_01155*	ABC transporter, permease protein	−2.71	0.02
*LMRG_01228*	*lmo2077*		*LMRG_01229-LMRG_01228*	Inactive homolog of metal-dependent proteases, Putative molecular chaperone tRNA (adenosine(37)-N6)-threonylcarbamoyltransferase complex dimerization subunit type 1 TsaB	−2.61	0.00
*LMRG_01283*	*lmo2129*			Hypothetical protein	−2.03	0.00
*LMRG_01286*	*lmo1681*	*metE*	*LMRG_01286-LMRG_01288*	5-methyltetrahydropteroyl-triglutamate–homocysteine methyltransferase	−2.23	0.00
*LMRG_01287*	*lmo1680*			Cystathionine gamma-synthase	−2.59	0.04
*LMRG_01333*	*lmo1633*	*trpE*	*trp*	Anthranilate synthase, aminase component	−2.21	0.00
*LMRG_01428*	*lmo1542*	*rplU*	*LMRG_01428-LMRG_01430*	50S ribosomal protein L21	−3.22	0.00
*LMRG_01435*	*lmo1535*		*LMRG_01435-LMRG_01436*	YebC/PmpR family DNA-binding transcriptional regulator	−2.25	0.00
*LMRG_01591*	*lmo2241*		*LMRG_01591-LMRG_01593*	Transcriptional regulator, GntR family	−2.25	0.00
*LMRG_01669*	*lmo2163*		*LMRG_01669-LMRG_01673*	Myo-inositol 2-dehydrogenase 1, Oxidoreductase	−2.21	0.00
*LMRG_01810*	*lmo2438*			ClbS/DfsB family four-helix bundle protein	−2.97	0.00
*LMRG_01955*	*lmo2741*		*LMRG_01955-LMRG_01957*	Multidrug-efflux transporter, major facilitator superfamily (MFS);Efflux pump Lde	−2.62	0.00
*LMRG_02234*	*lmo2688*		*LMRG_02235-LMRG_02233*	Cell division protein FtsW	−2.56	0.01
*LMRG_02481*	*lmo0052*		*LMRG_02481-LMRG_02483*	Phosphoesterase, DHH family protein	−2.07	0.00
*LMRG_02665*	*lmo0241*		*LMRG_02938*, *LMRG_02667-LMRG_02664*	TrmH family tRNA/rRNA methyltransferase YacO	−2.67	0.00
*LMRG_02671*	*lmo0235*	*ispD1*	*LMRG_02672-LMRG_02669*	2-C-methyl-D-erythritol 4-phosphate cytidylyltransferase	−2.27	0.00

### *LMRG_01669* and *LMRG_00091* Genes Were Differentially Expressed in ΔsigBCHL Compared to Wild Type Under BHI and Bile Exposure Conditions, Respectively

To further determine the role of σ^A^, the transcriptional profiles of the Δ*sigBCHL* mutant in BHI (control) and bile was used to compare to its wild type. We identified 369 and 357 genes that were differentially expressed in BHI or after bile exposure, respectively (Figures 2C,D). A total of 194 genes had statistically significantly lower transcripts levels in Δ*sigBCHL* mutant under BHI and/or bile conditions (Figure 2C). These genes were considered positively regulated by alternative sigma factors since we compared them with wild type. The *gadT2* gene, a member of *gadT2D2* operon encoding glutamate/gamma-aminobutyrate antiporter, was chosen to represent the genes in lower transcript group. The qPCR data showed that the level of *gadT2* expression was decreased in Δ*sigBCHL.* We identified 301 genes that were up-regulated in both or either conditions in Δ*sigBCHL*; 68 and 78 genes had higher transcripts in BHI alone or bile alone, respectively (Figure 2D). The up-regulated genes were defined as σ^A^-dependent genes since they showed higher expression levels in the quadruple mutant compared to wild type. Certainly, regulatory networks are more complicated. Lower expression levels in wild type could imply that these genes are negatively regulated in the presence of alternative sigma factors e.g., by the negative regulators that are not transcribed by σ^A^. For this study, we intentionally minimize discussions on negative roles of alternative sigma factors as de-repression of these genes (or enhanced levels of expression in Δ*sigBCHL*) was still σ^A^-dependent.

**FIGURE 3:**
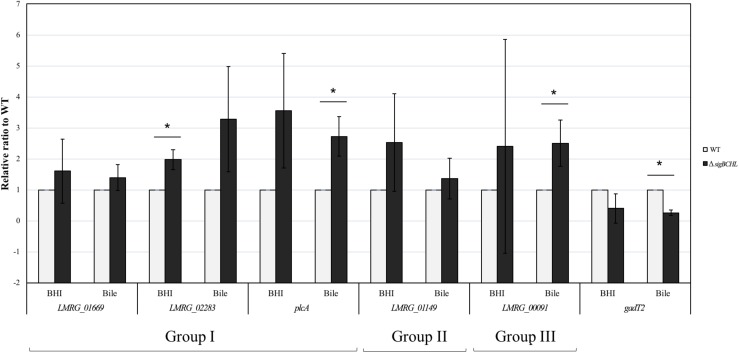
Confirmation of DEGs by TaqMan qPCR. Transcript levels were quantified in up-regulated sets of genes i.e., Group I (*LMRG_01669*, *LMRG_02283*, and *plcA*), Group II (*LMRG_01149*), and Group III (*LMRG_00091*) and in down-regulated gene (*gadT2*). Wild type transcript levels are in gray and Δ*sigBCHL* transcript levels are in black. Transcript levels are expressed relative to WT after normalization to geometric mean of housekeeping genes, *rpoB* and *bglA* (Supplementary Table S7). Experiments were performed in biological triplicates. Asterisk indicates significance fold difference relative to wild type.

**FIGURE 4:**
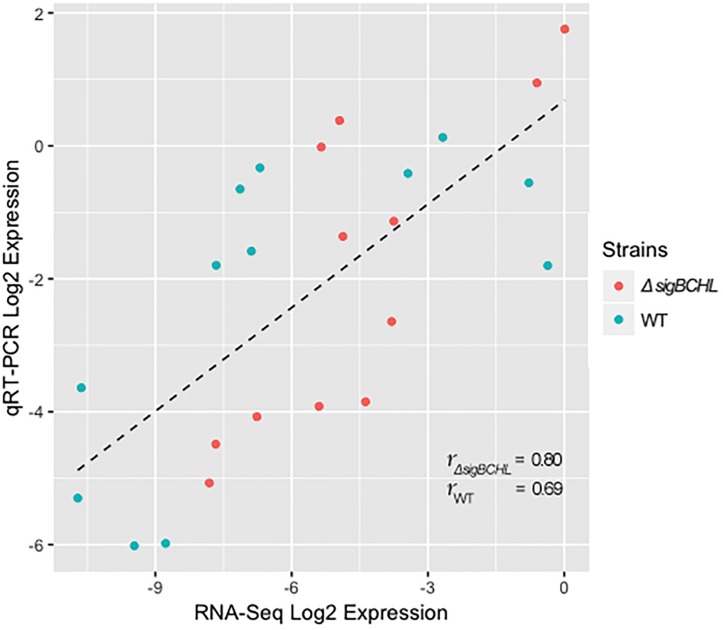
Pearson correlation between RNA-seq and qPCR for DEGs of *L. monocytogenes* wild type (blue) and quadruple mutant (red). All expression data were transformed to Log_2_ and normalized by geometric mean of housekeeping genes (*rpoB* and *bglA*).

We classified σ^A^-dependent genes into three groups: group I σ^A^-dependent genes expressed in both conditions, group II σ^A^-dependent genes expressed in BHI, and group III σ^A^-dependent bile inducible genes (Figure 2D). Among the 155 genes in group I, three of these genes (*LMRG_00092- LMRG_00094*) with higher FC are part of an operon that includes genes encoding a PTS fructose transporter subunit and alpha-mannosidase. *LMRG_02456* and *LMRG_02567* encode PTS system beta-glucoside-specific components. *LMRG_01933* encodes PTS system cellobiose-specific IIB component. *LMRG_01669-LMRG_01673* is an operon encoding genes involved in myo-inositol degradation and *LMRG_01535* is a phage capsid scaffolding encoded gene. *LMRG_02283* encodes membrane-spanning permease protein, MsmF, and a member of multiple sugar ABC transporter showed up to six FC. Known σ^A^-regulated genes in *prfA-plcA* operon are also present in this cluster (Supplementary Table S3). We then confirmed differential expression of *LMRG_01669*, *LMRG_02283*, and *plcA* in both BHI and bile conditions using qPCR (Figure 3). The RNA-seq and qPCR data correlate well with the Pearson correlation coefficients (*r*) of expression levels in the wild type and the quadruple mutant at 0.69 and 0.80, respectively (Figure 4). Expression of these genes are shown with higher transcripts in Δ*sigBCHL* than in WT in both BHI and bile conditions. Gene set enrichment analysis identified 48 GO terms that were overrepresented among group I members (Supplementary Table S4). They were mostly involved with cellular process, molybdopterin bioprocess, transmembrane transporter, and other transports.

Among the 68 genes that were up-regulated in the BHI control (group II), we found that *LMRG_01516* encoding for unknown protein showed the highest FC, as high as 52.6 (Supplementary Table S5). Other significantly up-regulated genes include an operon of ferrous iron transport genes (*LMRG_00057-LMRG_00059*), members of the *ilv* operon (i.e., *ilvB* and *ilvC*), and *LMRG_01149* encoding mannose specific IIC component in a PTS system. Higher transcript levels in *LMRG_01149* in BHI was validated by qPCR (Figure 3). Functional groups were enriched and significant specific GO terms associated with group II were not identified.

We identified 78 genes that were up-regulated only in bile exposure (group III; bile-stimulon) (Supplementary Table S6). *LMRG_00091* encoding PTS fructose transporter subunit IIA was up-regulated 27.7 FC. Beta-glucosidase encoding gene *LMRG_01934* as well as triosephosphate isomerase encoding gene *tpiA2* were up-regulated up to six FC. Interestingly, several bacteriophage-associated genes such as *LMRG_01524-LMRG_01525*, *LMRG_01536*, *LMRG_01540*, and *LMRG_01543* showed higher expression levels ranging from five to 14 FC. Virulence associated genes (*clpB*, *clpP*, *groL*, *inlB*, and *dnaK*) also had statistically increased their expression levels. However, GO terms associated with group III are not enriched.

## Discussion

A number of studies revealed the role of sigma factors in various stress responses using bioinformatic approaches including RNA-sequencing and protein level analyses. In this study, we define the exclusive role of housekeeping σ^A^ in BHI and under gallbladder bile (pH ∼8.2) stress exposure. In addition, we defined the bile stimulon, which includes genes involved mainly in stress defense mechanisms.

### Bile Mediates Up-Regulation of *dlt* and *ilv* Operons but Down-Regulated *prfA* and Heat Shock Genes

Approximately 2% of the total transcriptome was differentially expressed genes in *L. monocytogenes* 10403S wild type under bile exposure compared to BHI control. This finding showed less differentially expressed genes compared to a previous study (Guariglia-Oropeza et al., 2018) that showed approximately 16% of differentially expressed genes in *L. monocytogenes* 10403S exposed to bile pH 5.5. It has been shown that the toxicity of bile is pH-dependent; the lower the pH, the higher the toxicity (Begley et al., 2005b; White et al., 2015). As a result, the genes that respond to bile at lower pH would likely be different due to dual stresses. Nevertheless, the key differentially expressed genes are similar. For instance, *dltC* encoding D-alanyl carrier protein and *LMRG_02074* encoding teichoic acid D-Ala incorporation-associated protein DltX were induced under bile exposure. This finding supports that of others in that *dltABCD* operon is induced under bile exposure in both *L. monocytogenes* 10403S (lineage II) and H7858 (lineage I) (Guariglia-Oropeza et al., 2018) and *Lactobacillus rhamnosus* (Koskenniemi et al., 2011). DltABCD proteins are involved in D-alanylation of teichoic acid, which facilitates resistance to antimicrobial peptides (Revilla-Guarinos et al., 2014). These Dlt proteins have been shown to contribute to resistance to various cell wall disruption stress such as that induced by nisin (Kang et al., 2015) and lysozyme (Guariglia-Oropeza and Helmann, 2011). We also observed that *ilvBCD* genes were highly expressed in our bile exposure condition. The *ilv* operon is involved in branched-chain amino acid (BCAA) isoleucine or valine biosynthesis, which is essential for intracellular survival (Chatterjee et al., 2006). Recently, BCAA synthesis of isoleucine, leucine, and valine were shown to directly and indirectly affect PrfA (Lobel et al., 2012, 2015). Particularly, isoleucine is found to be a crucial signal for *L. monocytogenes* intra-host gene expression (Lobel et al., 2012; Brenner et al., 2018) and low concentrations of isoleucine can enhance virulence (Brenner et al., 2018). In addition to acting as signaling molecules, isoleucine and valine have been shown to serve as osmolytes that accumulate in the cytosol of plant cells under salt stress (Parida and Das, 2005). Bacteria accumulate osmoprotective solutes to manage hyperosmotic stress (Yancey et al., 1982). *L. monocytogenes* has been shown earlier to accumulate glycine, alanine, and proline that increase growth rate under high osmolarity (Patchett et al., 1992). Therefore, the induction of *L. monocytogenes ilv* operon under bile exposure may function as a signaling molecule pathway for accumulation of osmoprotectants that increase growth rate under high osmolarity.

We also found that *prfA* was down-regulated under bile exposure. This finding is consistent with previous microarray analysis showing that the entire PrfA regulon including *plcA*, *hly*, *mpI*, *actA*, *plcA*, *inlA*, and *inlB* was repressed in response to bile (Quillin et al., 2011). However, it conflicted with previous RNA-seq data in which some PrfA regulon genes were induced under bile exposure (Guariglia-Oropeza et al., 2018). This could be explained by the pH difference of the bile used in these studies. The microarray study used neutral bile while the RNA-seq used acidic bile pH 5.5. The bile components used in this study mimics gallbladder bile which is more similar to the condition used in the microarray study. The down-regulation of virulence associated genes such as *inlA* and *inlB* might allow the bacterium to initiate penetration after successful establishment (Prouty and Gunn, 2000), therefore conserving resources until primed for infection.

In agreement with previous proteomic analysis of *L. monocytogenes* under bile exposure, gene expression of chaperone proteins DnaK and DnaJ were lower upon bile treatment (Wright et al., 2016). Our data revealed that the expression of chaperone genes (i.e., *dnaK*, *groL*, and *groS*) were down-regulated under bile exposure in *L. monocytogenes* 10403S wild type strain. DnaK (heat-shock protein 70 family) and DnaJ (heat-shock protein 40 family) are involved in protein folding and survival under stress conditions (Genevaux et al., 2007). GroL exists in a double heptameric ring structure, which facilitates newly synthesized proteins folding. Deletions of *dnaK* in *Campylobacter jejuni* and *Salmonella enterica* serovar Typhimurium lead to diminish growth in macrophages and reduced ability to colonize mice (Konkel et al., 1998; Takaya et al., 2004). Heat shock proteins are important for bacterial survival in host niches (Neckers and Tatu, 2008). However, inactivation of *hspR* genes (encoding heat shock protein receptor and transcription factor) in *Mycobacterium tuberculosis* yielded high production of DnaK resulting in significantly impaired persistence in the mouse model (Stewart et al., 2001; Das Gupta et al., 2008). Findings in other organisms suggest that increased DnaK protein concentrations could boost the immune response in early stages of infection and possibly lead to expedited clearance of the bacteria. These data suggest that overexpression of the heat shock proteins might not increase the virulence of the bacteria. Deviant production of the heat shock proteins is possibly disadvantageous to the pathogen and regulating the magnitude and the timing of the production are important. Moreover, in *E. coli* and, DnaK inhibits the σ^32^ or σ^*H*^ by binding to and targeting it for degradation via the FtsH, a membrane-bound protease (Tomoyasu et al., 1995; Blaszczak et al., 1999). They found that the induction of σ^32^ in DnaKJ depleted-cell induces broad changes in proteomes and re-organization in cellular function in which, the majority of repair and maintenance functions are up-regulated, while the proliferation and metabolic process go down. Therefore, it suggests that DnaK is not only involved in the DNA replication or misfolded protein catalytic, but also essential for regulatory function when its folding activity is dispensable (Schramm et al., 2017). Collectively, the down-regulation of *dnaK*, *groL*, and *groS* genes after bile exposure in our study may support avoidance of the immune system in early stage of infection as well as play a part in a regulatory complex for maintenance activity.

### σ^A^ Compensates Functions of Other Alternative Sigma Factors

As the quadruple alternative sigma factor mutant had a comparable phenotype to wild type under bile exposure, we queried differentially expressed genes in the quadruple mutant under BHI and bile exposure. Surprisingly, only 29 genes were differentially expressed between the *L. monocytogenes* quadruple mutant exposed to bile compared to BHI control. Although it has no alternative sigma factors, the number of differentially expressed genes was limited. It is feasible that σ^A^ compensated for the alternative sigma factors, thus resulting in a small number of differentially expressed genes. One possible explanation could be the competition among sigma factors. The concept of “competition” sets in when the concentrations of sigma factors are in excess of core RNAP (Mauri and Klumpp, 2014). The competition is more complicated when the affinities of different sigma factors are varied. The stronger-binding sigma factor dislocates the lower affinity sigma factors even under concentration equilibrium of alternative sigma factors. Previous study has measured *E. coli* sigma factors dissociation constants and revealed that the housekeeping sigma factor σ^70^ is found to have the strongest-binding affinity to core RNAP (Maeda et al., 2000). In addition, the affinity of the housekeeping sigma factor binding to core RNAP can be modulated by alarmone ppGpp (guanosine tetraphosphate), which is induced in bacteria or plants by several stresses (Srivatsan and Wang, 2008). If the ppGpp can specifically modulate housekeeping sigma factors, but not other alternative sigma factors, then during stress ppGpp can enhance successful competition of alternative sigma factors over the housekeeping (Mauri and Klumpp, 2014). Therefore, in the absence of alternative sigma factors in the quadruple mutant, no competition can be observed leading to enhanced function of the housekeeping sigma factor σ^A^.

Moreover, it has previously been shown that strong binding between promoter and RNAP could lead to relatively lower transcription initiation rates since the promoter is occupied by RNAP most of the time (Hatoum and Roberts, 2008). For example, in *E. coli*, σ^*N*^ which is structurally and mechanically different from other sigma factors (Mauri and Klumpp, 2014) together with core RNAP can occupy a promoter sequence and, upon binding to RNAP holoenzyme, the promoter remains inactive in a closed transcription initiation complex. The complex becomes active upon binding of the ATPase activator, which typically occupy a site at a distance from the promoter and contacts the holoenzyme via DNA looping (Friedman and Gelles, 2012). In *L. monocytogenes*, σ^*L*^ is classified as a member of RpoN (σ^54^), which is structurally similar to σ^*N*^ in *E. coli*. Thus, existence of alternative sigma factor such as σ^*L*^ could passively prevent transcription that would otherwise be initiated by other sigma factors resulting in a temporary stop in transcription. In addition, the cross-talk among alternative sigma factors take place in various regulatory networks. The quadruple mutant with no alternative sigma factors would not have the insulation effect, therefore, it drives transcription more conveniently.

### σ^A^-Dependent Genes

Most of the σ^A^-dependent genes are considered as housekeeping genes that are important for the growth of the bacteria. It is equally important to better understanding the role of σ^A^ during stress exposures. Here, we report the list of σ^A^-dependent genes that were higher expressed in both BHI and bile conditions to provide the information of σ^A^-dependent and housekeeping genes for further analyses. The functional categories of σ^A^-dependent genes in this study using gene set enrichment analysis (GOseq), were found that these genes are involved in many crucial biological processes including carbohydrate metabolic process, pentose phosphate shunt, NADP metabolic process, transport, cell communication, signal transduction and regulation, which are important for cell growth and survival.

The *bglA* gene encoding beta-glucosidase is used as a reference housekeeping gene in several studies (Tasara and Stephan, 2007; Kwong et al., 2016; Hilliard et al., 2018) and is shown in our list. The level of *bglA* was constantly expressed in both conditions. In addition to *bglA*, the entire five genes in a σ^A^-dependent operon *LMRG_01669-LMRG_01673* are shown in our list as constantly expressed at high levels in both conditions. This operon is involved in myo-inositol degradation. In the Gram-negative bacterium, *Legionella pneumophila*, myo-inositol promotes its growth and virulence for infection of amoeba and macrophage (Manske et al., 2016). Our qPCR confirmed that the expression level of the representative gene in this operon, *LMRG_01669* was constantly expressed in both conditions. Therefore, this gene can be used as a σ^A^-dependent gene in further study.

### σ^A^-Dependent Bile Inducible Genes

Since acidic bile (pH 5.5) does not induce the σ^B^ regulon, we further focused on the housekeeping sigma factor σ^A^ regulation upon basic bile exposure (pH 8.2). Consistent with previous bile stimulon findings, we confirmed that *LMRG_01119* encoding a PTS system enzyme in the *LMRG_01117 – LMRG_01121* operon is a bile responsive gene. Here, we reported that this lineage II specific gene is σ^A^-dependent and induced by bile. This operon is suggested to facilitate bacterial survival during gastrointestinal stages of infection (Guariglia-Oropeza et al., 2018). Besides *LMRG_01119*, *LMRG_00091* encoding for PTS fructose transporter subunit is σ^A^-dependent bile inducible gene with high FC ∼ 27. In contrast, the PTS associated proteins in either glucose or mannose systems from previous proteomic study have decreased upon exposure to bile (Wright et al., 2016). To better understand the link between them, further investigation is warranted.

The up-regulation of *clpP*, a gene involved in degradation of misfolded proteins and required for growth subsequent to stress exposure, was consistent with previous reports demonstrating the level of ClpP protein increased in *L. monocytogenes* HCC23 strain under bile exposure in aerobic condition (Gaillot et al., 2000; Wright et al., 2016). This data suggested that *clpP* is induced in response to damaged proteins; bile contributes to conformational changes of proteins resulting in misfolding and denaturation (Begley et al., 2005a). Importantly, the induction of *inlB*, encoding the surface protein of *L. monocytogenes* that partially mediates entry into host cell by binding to the host receptor Met (Bierne and Cossart, 2002), was observed. It can implicate that bile triggers expression of bacterial genes involved in host cell invasion.

In contrast to differentially expressed genes in *L. monocytogenes* wild type, we found that the chaperone genes, *dnaK* and *groL* were σ^A^-dependent and induced under bile exposure. Due to the lesser complicated regulatory network in quadruple mutant, regulated by σ^A^, the negative effects of other alternative sigma factors such as insulation effects on promoter sequences were removed; therefore, the up-regulation of these chaperone genes was noticed. It could suggest that σ^A^ drives the expression of *dnaK* and *groL* in response to bile, hence, we conclude that these genes are σ^A^-dependent bile inducible genes.

## Conclusion

In this study, we used RNA-seq to define transcriptomes of *L. monocytogenes* 10403S wild type and its isogenic quadruple mutant (Δ*sigBCHL*) in BHI and under 1% gallbladder bile (pH 8.2) exposure. This study underscores that σ^A^ is a housekeeping sigma factor that can compensate for the absence of all other sigma factors. Our data suggest σ^A^ is sufficient to survive in gallbladder bile. The σ^A^-dependent bile inducible genes are represented by PTS systems, chaperones, and transporters, which contribute to cellular homeostasis of the bacteria. We proposed a compensatory role of σ^A^ in the absence of alternative sigma factors.

## Data Availability

The datasets generated or analyzed for this study can be found in the sequence read archive (SRA): https://www.ncbi.nlm.nih.gov/sra/PRJNA544468.

## Author Contributions

HO and SC conceived the study. AB performed the experiments and analyzed the data. All authors drafted and approved the final version of the manuscript.

## Conflict of Interest Statement

The authors declare that the research was conducted in the absence of any commercial or financial relationships that could be construed as a potential conflict of interest.
